# Phenotypic and molecular characterization of clinical isolates of *Acinetobacter baumannii* isolated from Delhi, India

**DOI:** 10.1186/s12941-015-0101-5

**Published:** 2015-09-04

**Authors:** Dabet Rynga, Malini Shariff, Monorama Deb

**Affiliations:** Department of Microbiology, Vallabhbhai Patel Chest Institute, University of Delhi, Delhi, 110007 India; Vardhaman Mahavir Medical College and Safdarjung Hospital, New Delhi, 110029 India; Department of Microbiology, Vardhaman Mahavir Medical College and Safdarjung Hospital, New Delhi, India

**Keywords:** *Acinetobacter baumannii*, Molecular characterization, MLST, RAPD, Beta-lactamases

## Abstract

**Background:**

Acinetobacter has gained importance as a multi-drug resistant and hence a difficult to treat pathogen. This study was done to characterize our isolates with respect to drug resistance and presence of beta-lactamases which is a major mechanism of resistance and to type using RAPD and MLST so that comparison of our clones can be made with the existing international clones.

**Methods:**

100 isolates recovered from clinical samples from two hospitals in Delhi were tested for their susceptibility against major groups of antimicrobials. The resistant isolates were screened and confirmed phenotypically for presence of ESBL, MBL and AmpC and MBLs also by PCR. The isolates were typed by RAPD and MLST.

**Results:**

Out of the 100 isolates, 91, 78 and 2 % were MDR, XDR and PDR respectively. 97, 100 and 85 were screen positive for ESBL, AmpC and MBL respectively. Of these, 38.1 % were confirmed phenotypically to produce ESBL, 99 % produced AmpC and 29.4 % produced MBL comprising of GIM, VIM, SIM and IMP. MLST showed known STs 110, 188, 146, 69, 103, 108 and 194. Eight new STs were encountered. The RAPD showed a high degree of genetic variability among the isolates.

**Conclusion:**

Majority of our isolates were MDR, producing one or more types of beta-lactamases. We encountered drug resistant international clones by MLST which are found in other continents there by confirming their spread to Indian sub continent. No data on ST types of other Indian isolates is available in the MLST database and hence comparison is not possible.

## Background

*Acinetobacter* species, once considered as opportunistic, low virulence pathogens have now emerged as important nosocomial pathogens due to their increase in antimicrobial resistance. The resistance is due to various mechanisms including production of different types of beta-lactamases including oxacillinases. They are responsible for a number of hospital acquired infections. To control the spread of *Acinetobacter baumannii* (*A. baumannii*) in the hospital, it is necessary to distinguish the outbreak strain from epidemiologically unrelated *Acinetobacter.* This requires the comparison of isolates at the subspecies level which is done by epidemiological typing methods. Phenotypic typing systems based on biochemical profiles (biotyping), antibiotic susceptibility patterns, serological reactions (serotyping), phage typing and protein profiles have mostly been replaced by molecular typing systems like ribotyping, Pulsed-field gel electrophoresis (PFGE), Amplified fragment length polymorphisim (AFLP) analysis, Random amplification of polymorphic DNA (RAPD) and many more. PFGE is more sensitive in analysis of *Acinetobacter* epidemiology but it is cumbersome as well as expensive, so RAPD is preferred. However, reproducibility and inter-laboratory exchange of data for global epidemiological analysis have been problematic which is solved by MLST by offering the possibility to transfer typing data from laboratory to laboratory or compare results via the internet [1].

The present study was undertaken to identify and characterize clinical isolates of *A. baumannii* with reference to its antibiotic susceptibility, presence of the different types of β-lactamases in the resistant isolates and molecular typing of the isolates using RAPD and MLST and compare them with international clones.

## Methods

Institutional ethics committee clearance was taken before the start of the project. A total of one hundred (100) non repetitive isolates of *A. baumannii* were collected from various clinical specimens from patients attending two hospitals in New Delhi after taking patient’s consent. The species identification of the isolates was done using the API 20NE strips (Biomerieux, Cat No. 20 050) and it was further confirmed using Amplified ribosomal DNA restriction analysis (ARDRA) [2, 3].

Susceptibility of the isolates to various antibiotics was tested by using the Kirby Bauer’s Disk diffusion method. The antibiotics used included cefoxitin (30 μg), ceftazidime (30 μg), cefotaxime (30 μg), cefpodoxime (30 μg), aztreonam (30 μg), cefepime (30 μg), imipenem (10 μg), amikacin (30 μg), ciprofloxacin (30 μg), gentamicin (10 μg), ampicillin/sulbactam (20/10 μg), piperacillin/tazobactam (100/10 μg), tigecycline (15 μg) and colistin(10 μg) discs (Becton–Dickinson). The diameters of the zones of inhibition were recorded and interpreted as sensitive, intermediate sensitive or resistant, according to the CLSI guidelines 2013 [4] except colistin and tigecycline where in CLSI guidelines for *Acinetobacter* is not available. Keeping the breakpoints of ≤2 as sensitive and ≥4 as resistant [5] the zone sizes of colistin in disk diffusion test was taken as ≥11 as susceptible and ≤10 as resistant [6, authors unpublished data]. The interpretation for tigecycline was ≥16 mm as sensitive and ≤12 as resistant according to Jones et al. [7]. *A. baumannii* isolates were labelled as Multi-drug resistant (MDR) if the isolate was resistant to at least three classes of antimicrobial agents—all Penicillins and Cephalosporins (including inhibitor combinations), Fluroquinolones, and Aminoglycosides, Extensive drug resistant (XDR) when they were MDR and also resistant to Carbapenems and finally, Pan-drug resistant (PDR), those that are XDR and also resistant to polymyxins and tigecycline [8].

The isolates were screened for ESBL production by checking their susceptibility against ceftazidime, cefotaxime, cefpodoxime and aztreonam (each disk of 30 μg) and the screen positive isolates were confirmed phenotypically by the Modified combined disc test in which AmpC inhibitory substances, like cloxacillin (200 μg/ml) is added into the Mueller–Hinton Agar as the coexistence of AmpC β-lactamases along with ESBL have been shown to mask the production of ESBL. If the zone of inhibition around cefotaxime-clavulanate is larger than the zone around the cefotaxime disc then the isolate is ESBL positive [9].

Isolates resistant to imipenem and ceftazidime were further confirmed for MBL production by Combined disc test. An organism was considered to be MBL positive if there was an increase of ≥7 mm in the zone of inhibition around the imipenem + EDTA disc as compared to imipenem disc alone [10]. They were further confirmed for the presence of MBL genes, VIM, IMP, GIM, SPM and SIM, using multiplex PCR [11].

Similarly, isolates resistant to cefoxitin were confirmed phenotypically for the presence of AmpC β-lactamase by the AmpC disc test. Briefly, a lawn culture of cefoxitin sensitive *E.coli* (ATCC 25922) was prepared on Muller Hinton Agar plate and a cefoxitin disk (30 μg) was placed on it. A sterile plain disk was placed next (almost touching) to the cefoxitin disk, moistened with 20 μl of sterile saline and inoculated with several colonies of the test organism. A flattening or indentation of the cefoxitin inhibition zone in the vicinity of the inoculated disc was considered as a positive test [12].

Typing of the isolates was done using RAPD and MLST methods.

RAPD was performed by the method described by Karthika et al. 2009 [13]. Amplification was carried out in a final volume of 10 μl of the amplification mixture containing 1U of *Taq* Polymerase, 1X of PCR buffer (with MgCl2), 50 pmol of primer, 50 μM of deoxynucleotide triphosphate (dNTP) and 1 μl of DNA template. AP6 CCCGTCAGCA was used as a primer. 35 cycles of 94 °C for 30 s, 45 °C for 45 s, 72 °C for 2 min followed by a final extension 72 °C for 5 min. The PCR products were resolved using 1.2 % agarose gel. A low molecular weight DNA ladder (New England Biolabs Inc., Category no. N3233S) was used. The image of the gel was captured and the banding pattern was analyzed using Gelcompar II software (Applied-Maths, Kortrijk, Belgium) and the dendrogram was generated.

For MLST, seven housekeeping genes were amplified using the method described by Bartual et al., [14] with some modifications in the sequence of *gyrB* and *rpoD* primers as given by Park et al. [15]. The PCR products were resolved by 1 % agarose gel electrophoresis. Amplified fragments were sequenced by outsourcing to a commercial company. The sequences obtained were trimmed using the Bio Edit Sequence alignment editor software (Ibis Biosciences, Carlsbad, CA, USA). The assignment of alleles and sequence types was performed by the software available in the Department of Zoology, Oxford University website noted above. The sequence types not present in the database were submitted to the website and assigned new ST types.

## Results

Out of the 100 isolates of *A. baumannii* 14 % were isolated from sputum, 31 % from ET aspirate, 28 % from pus, 25 % from wound swab, 1 % from drain fluid and 1 % from high vaginal swab. *A. baumannii* was isolated from ICU (28 %), followed by burns ward and respiratory medicine ward (15 % each), surgery ward (14 %) burns ICU (11 %) gynaecology ward (9 %), orthopaedics ward (5 %), respiratory medicine OPD (2 %). Respiratory samples were from patients suffering from exacerbation of Chronic obstructive pulmonary disease, Asthma, Chronic bronchitis and Ventilator associated pneumonia.

The results for detection of *A. baumannii* by ARDRA (using three enzymes) and API 20 NE strips were identical.

The antibiotic susceptibility of the isolates is as shown in Table 1. High percentage of resistance was seen to most antibiotics except colistin and tigecycline.

**Table 1:** Antibiotic susceptibility pattern of *A. baumannii* isolates (n = 100)

Antibiotics	Resistant (%)	Intermediate (%)	Susceptible (%)
Ceftazidime	99 (99)	1 (1)	0 (0)
Cefotaxime	100 (100)	0 (0)	0 (0)
Cefpodoxime	100 (100)	0 (0)	0 (0)
Aztreonam	99 (99)	1 (1)	0 (0)
Imipenem	85 (85)	5 (5)	10 (10)
Cefoxitin	100 (100)	0 (0)	0 (0)
Amikacin	97 (97)	0 (0)	3 (3)
Ciprofloxacin	98 (98)	1 (1)	1 (1)
Gentamicin	95 (95)	1 (1)	4 (4)
Piperacillin–tazobactam	85 (85)	8 (8)	7 (7)
Cefepime	100 (100)	0 (0)	0 (0)
Ampicillin–sulbactam	65 (65)	14 (14)	21 (21)
Tigecycline	42 (42)	–	58 (58)
Colistin	3 (3)	–	97 (97)

Out of the 100 isolates, 91 % were MDR, 78 % were XDR and 2 % were PDR.

Screening tests showed 97 (97 %), 100 (100 %) and 85 (85 %) were positive for ESBL, AmpC and MBL respectively. Out of these screen positive isolates, 37 (38.1 %), 99 (99 %) and 25 (25 %) were confirmed phenotypically to produce ESBL, AmpC and MBL respectively. 18 out of the 25 isolates that were confirmed phenotypically for MBL production, were positive for GIM (n = 6), VIM (n = 9), SIM (n = 2) and IMP (n = 1) by PCR. AmpC was seen to be produced by all but one isolate. It existed in combination with ESBL in 37 isolates and with MBL in 25. Interestingly, 7 isolates showed the production of all three.

**Fig. 1:**
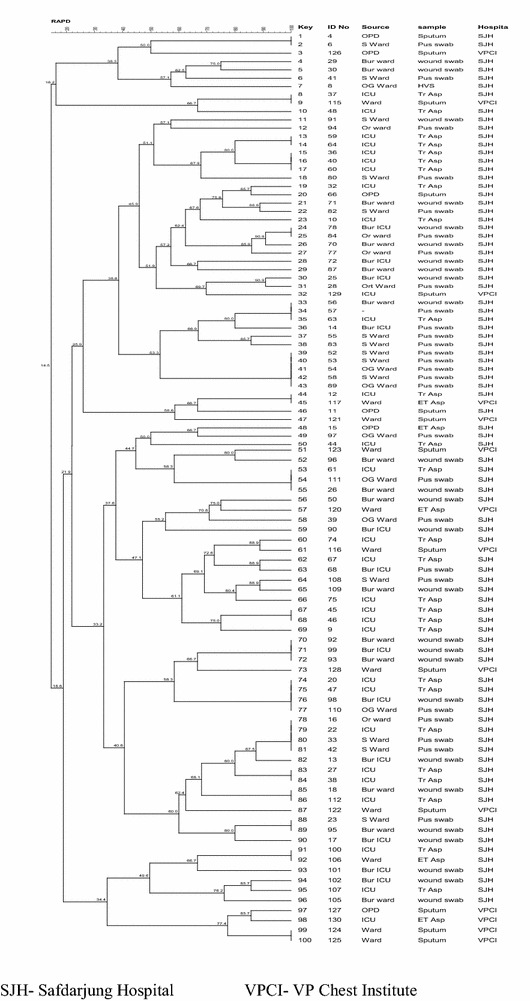
UPGMA clustering dendrogram indicating percentage similarity between RAPD patterns of *A. baumannii* isolates from Safdarjung hospital and VPCI

Total of 100 isolates were typed using RAPD. 86 isolates were from one hospital and 14 from the other. Figure 1 shows the dendrogram of the isolates showing percentage similarity between them. A high degree of genetic variability was observed among the 100 isolates, including 53 distinct RAPD patterns with >20 % difference in UPGMA generated dice coefficients. 18 of these showed 100 % similarity. Majority of the isolates in each clonal type were restricted to the same hospital. However, few clonal types showed isolates from both the hospitals. The discriminatory index of 0.984 was found to be good.

MLST was done on 21 isolates representative of different clones as indicated by RAPD. One isolate each of STs 110, 103, 108, 194 and 14, two isolates each of ST 146 and 69 and three isolates of ST 188 were encountered. Nine isolates were found to have new ST types and were submitted to the PUBMLST site and were assigned the types. The new STs assigned were, ST 386 (n = 1), ST 387 (n = 1), ST 388 (n = 1), ST 389 (n = 1), ST 390 (n = 2) and ST 391 (n = 3) (Table 2).

**Table 2:** Sequence types of *Acinetobacter baumannii* isolates encountered in the study

S. no	ST	gltA	gyrB	gdhB	recA	cpn60	Gpi	rpoD	Strain
1	386*	31	33	67	40	1	102	7	In-S 58938
2	387*	1	15	4	11	4	140	4	In-S 95167
3	388*	1	15	4	11	4	95	4	In-S 91318
4	389*	1	7	8	11	1	4	14	In-S 93192
5	390*	31	33	67	40	1	58	7	In-S 100924
6	391*	1	15	13	12	4	102	2	In-V 1687/11
7	391*	1	15	13	12	4	102	2	In-V 323/05
8	391*	1	15	13	12	4	102	2	In-V 5930/11
9	390*	31	33	67	40	1	58	7	In-V 5290/10
10	14	1	10	8	6	1	4	14	In-S 84570
11	103	12	17	12	1	29	3	39	In-S 90868
12	69	1	46	3	2	2	58	3	In-S 98632
13	69	1	46	3	2	2	58	3	In-S 101149
14	108	10	12	4	6	4	9	5	In-S 101767
15	194	1	15	4	11	4	58	4	In-S 102704
16	110	1	15	2	28	1	52	32	In-V 5290/08
17	188	31	33	67	40	16	58	7	In-V 7362/07
18	188	31	33	67	40	16	58	7	In-V 3507/06
19	188	31	33	67	40	16	58	7	In-S 88877
20	146	1	15	13	12	4	14	2	In-V 8095/10
21	146	1	15	13	12	4	14	2	In-S 92729

*S.no.* serial number

* New STs found in the study

## Discussion

The distribution of the isolates among the specimen and the wards were similar to a recent study done in North India where it was seen that maximum number of *Acinetobacter* were isolated from pus (37.14 %), followed by blood (23 %) and urine (13.6 %). The highest percentage of isolation was from the ICU (22 %), followed by paediatrics (21 %), neurosurgery (16 %) and general surgery ward (13 %) [16].

When compared with other Gram-negative bacilli, the outer membrane of *A. baumannii* is less permeable which might be due to the small number and size of porins and thus it is intrinsically less susceptible to antimicrobial agents [17].

The resistance of *A. baumannii* isolates to Cephalosporins was similar to the findings of studies done in Saudi Arabia and Nigeria where in the resistance was found to range from 70 to 100 % [19] which might be due to the extensive use of Cephalosporins in these hospitals.

The resistance to the Aminoglycosides—amikacin and gentamicin was similar to the results in two studies where it was found to be 78.2–79.5 and 96.2 % respectively [20, 21]. In contrast, a few studies which were carried out in China and Malaysia showed that it was as low as 20.5–57.4 and 66.7 % respectively, which might be due to the controlled use of Aminoglycosides in these hospitals [22, 23].

The resistance to Fluroquinolone, like ciprofloxacin, was similar to a study from Egypt which showed the resistance to be 85 % [24].

After the development of resistance to Fluoroquonolones, Carbapenem is usually the drug of choice for infections caused by Multi-drug resistant *A. baumannii* but unfortunately, Carbapenem-resistant isolates are constantly on the rise. In the present study, the resistance to imipenem (85 %) was similar to the findings from other parts of the world. [25–27]. In contrast three studies, two from India and one from Saudi Arabia, showed the resistance to be still as low as 0 and 9 % respectively. This might be due to the small number of isolates tested in these studies. [28–30].

With the increase in Carbapenem-resistance, tigecycline and colistin have been used for treatment of the infections caused by Extensive-drug resistant (XDR) *A. baumannii*.

There is no CLSI guidelines for the disk diffusion criteria for *A. baumannii*. United States Food and Drug Administration breakpoint criteria for tigecycline when testing Enterobactericeae (Susceptibility at ≤2 μg/ml, intermediate at 4 μg/ml and resistant at ≥8 μg/ml and the corresponding disc diffusion diameters as ≥19 and ≤14 mm for sensitive and resistant strains respectively) is being followed by various authors. But it was observed by Jones et al., that using these zone sizes many isolates were falsely labelled as resistant [7]. She proposed that by using the zone diameters as ≥16 mm for sensitive and ≤12 mm for resistant isolates reduced the error rate to a minimum. Thus, using these criteria, in the present study, resistance to tigecycline was found to be 42 % which was similar to a study done in Taiwan in which it was 45.5 % [31]. But, it was very high in comparison to a study by Behera et al. [32] in which, in spite of using the FDA breakpoints, the resistance was only 7.6 %. This could be due to the fact that the study was conducted in 2007 when tigecycline was not used as frequently as Carbapenems for the treatment of these infections. Resistance to tigecycline has also increased in the past few years [33].

In this study, the resistance to colistin was similar to that seen in three other studies, one each from India, Egypt and Taiwan which showed a resistance of 3.5, 5 and 10 % respectively [18, 22, 30]. This might be due to the fact that colistin is a reserve drug and used only for multi-drug resistant cases. However, a recent study from the United States showed that 14 out of 28 isolates were colistin resistant [34]. This is because of the rise in MDR *Acinetobacter* where in, colistin is the only choice available for the treatment. Hence, resistance to it is also emerging. The resistance to antibiotics reflects on the policy of antimicrobial usage and the circulation of drug resistant clones in different countries.

In the present study it was seen that out of the 100 isolates, 91 % were MDR, 78 % were XDR and 2 % were PDR. Similar findings were seen in another study. [35]. However, a study from North India showed lower percentage of isolates to be MDR. [20]. This could be due to the fact that the study was done in 2007–2008 and usage of Carbapenems and hence, resistance to it has increased since then.

The resistance to the various antibiotics is commonly due to the production of β-lactamases out of which the Extended spectrum β-lactamse (ESBL), AmpC β-lactamase and the Metallo β-lactamase (MBL) were studied here. All these three mechanisms have been observed in the present study.

Detection of lower number of MBLs by PCR is due to the fact that MBLs are only one of many mechanisms for Carbapenem resistance. Oxacillanases are important cause of Carbapenem resistance in *Acinetobacter*. Unfortunately we could not test for it.

In the present study, 99 % of the isolates produced AmpC, 37 % in combination with ESBL and 25 % in combination with MBL. Co-production of all the three, ESBL, AmpC and MBL were found in 7 % of our isolates. This could explain the increased rates of MDR in our study.

RAPD was done to detect clones which could be further typed by MLST. In the current study RAPD showed a high degree of genetic variability among the 100 isolates, with 53 distinct RAPD patterns out of which 18 (consisting of 2–5 isolates each) showed 100 % similarity between the isolates. The clonal types were not restricted to specific wards but were spread all over. They seem to be mostly restricted to the same hospital though a few were seen in both hospitals. Interestingly, one clonal type comprising of 5 isolates isolated from tracheal aspirates of different patients in the ICU of SJH, were Extensive-drug resistant, resistant to tigecycline, sensitive to colistin. Another clonal type included 6 isolates from different samples and different wards of SJH but all were Extensive-drug resistant, sensitive to colistin and all but one were sensitive to tigecycline. There were many other clonal types but they did not correlate with the antibiotypes. In a study done in India it was seen that the RAPD results did not correlate with those of antibiotypes since the isolates showed highly divergent resistance profiles. Only a few correlations could be made [13].

PUBMLST database has 2738 *Acinetobacter baumannii* isolates with 920 assigned STs. Analyzing the data by eBURST, 78 groups (each having 6 or more loci in common) were identified. Out of all the STs identified 319 were singletons not belonging to any group. Even among the 78 groups a founder could only be identified in very few suggesting high diversity among the isolates (http://pubmlst.org/abaumannii/). Few of the STs found in the study with clonal complex 92, 104, 109, 110 and 20 belonged to international clones and are distributed in different continents of the world suggesting their spread to India due to frequent travel between the continents. Most of these clones are multidrug resistant and that could explain the increased percentage of MDR in our study. So far, there is no published data on the sequence types of other Indian isolates. MLST database does not contain ST of Indian isolates. Therefore, the prevalence of the ST types in India cannot be commented upon.

In comparison of ST types with the antibiotic resistance pattern it was observed that, ST146 (CC20) clone consisting of 2 isolates which were XDR and sensitive to both tigecycline and colistin, did not produce ESBL or MBL but produced AmpC β-lactamase, were from different clinical samples and different wards. CC20 is an international clone and is known to produce Oxa 51- and 68-variants. This could explain the XDR nature of our isolates though we did not test for oxacillinases. Two other isolates belonged to another clone ST69 and they were both XDR, resistant to tigecycline and sensitive to colistin, producing both ESBL and AmpC β-lactamase but they belonged to different wards. ST110 clone consisted of two isolates which although had different antibiotic susceptibility, they produced the same type of β-lactamase. ST188 clone with three isolates showed similar antibiotic susceptibility but differed in the type of β-lactamase produced. The discordance between the ST types and antibiotic resistance and/or the β-lactamase types could be due to the fact that MLST is based on the house keeping genes but the genes responsible for resistance and virulence could be located on mobile elements and clustered in genomic islands and are not linked to particular sequence types. The new STs encountered in our study were mostly sensitive to colistin and resistant to tigecycline. They were closely related and were either single locus variants or double locus variants of existing clones. For example ST 390 is a SLV and 386 a DLV of known ST 188. These two differed from 188 in that both were resistant to tigecycline. All of these were isolates from respiratory samples. Similarly ST 387 and 388 were SLV and DLV of ST 194 respectively. All these isolates were from burns unit of the same hospital. Two of the three were resistant to tigecycline. These data show that genetic variations have occurred locally in the existing clones to give the new STs.

## Conclusion

A high percentage of MDR and XDR *Acinetobacter* obtained in the study is due to acquisition of various kinds of beta lactamases most of these belonging to resistant international clones. Hence, *A. baumannii* has developed into one of the most difficult hospital pathogens to control and treat. As of now colistin is the only drug of choice due to its low resistance. But keeping in mind the nephrotoxicity, patients should be carefully treated giving adequate dosage and proper scheduling. Restrained and careful use of antibiotics as well as strict infection control policy is crucial for preventing the emergence of complete resistance and spread of this pathogen. RAPD with a discriminatory index of 0.984 can be used to type isolates. MLST should be performed for epidemiological surveillance so as to learn about the variability and prevalence of MDR clones in the world.

## References

[1] Peleg AY, Seifert H (2008). Paterson DL *Acinetobacter baumannii*: emergence of a successful pathogen. Clin Microbiol Rev.

[2] Chandra R, Kapil A, Sharma P, Das B (2002). Identification of *Acinetobacter* species isolated from clinical specimens by amplified ribosomal DNA restriction analysis. Indian J Med Res.

[3] Vaneechoutte M, Dijkshoorn L, Tjernberg I, Elaichouni A, de Vos P, Claeys G, Verschraegen G (1995). Identification of *Acinetobacter* genomic species by amplified ribosomal DNA restriction analysis. J Clin Microbiol.

[4] Clinical Laboratory Standards Institute (CLSI). Performance standards for antimicrobial susceptibility testing. Twenty-Third Informational Supplement (M100-S23), Wayne, PA.2013.

[5] Wikler MA (2008). Performance standards for antimicrobial susceptibility testing; Eighteenth informational supplement.

[6] Somily AM (2010). Comparison of E-test and disc diffusion methods for the in vitro evaluation of the antimicrobial activity of colistin in multi-drug resistant Gram-negative bacilli. Saudi Med J.

[7] Jones RN, Ferraro MJ, Reller LB, Schreckenberger PC, Swenson JM, Sader HS (2007). Multicenter studies of tigecycline disk diffusion susceptibility results for *Acinetobacter spp*. J Clin Microbiol.

[8] Manchanda V, Sanchaita S, Singh NP (2010). Multidrug resistant *Acinetobacter*. J Global Infect Dis.

[9] Thomson KS (2010). Extended-spectrum-beta-lactamase, AmpC, and carbapenemase issues. J Clin Microbiol.

[10] Yong D, Lee K, Yum JH, Shin HB, Rossolini GM, Chong Y (2002). Imipenem-EDTA disk method for differentiation of metallo-beta-lactamase-producing clinical isolates of *Pseudomonas* spp. and *Acinetobacter* spp.. J Clin Microbiol.

[11] Ellington MJ, Kistler J, Livermore DM, Woodford N (2007). Multiplex PCR for rapid detection of genes encoding acquired metallo beta-lactamases. J Antimicrob Chemother.

[12] Black JA, Moland ES, Hossain A, Lockhart TJ, Olson LB, Thomson KS (2003) Abstr. 43rd intersci. Conf. Antimicrob. Agents Chemother., abstr C2-2034. Prevalence of plasmid-mediated AmpC β-lactamase in *Klebsiella pneumoniae* (kp), *Klebsiella* spp. and 21 non-ICU sites in the United States.

[13] Karthika RU, Rao RS, Sahoo S, Shashikala P, Kanungo R, Jayachandran S (2009). Prashanth K Phenotypic and genotypic assays for detecting the prevalence of metallo-beta-lactamases in clinical isolates of *Acinetobacter baumannii* from a South Indian tertiary care hospital. J Med Microbiol.

[14] Bartual SG, Seifert H, Hippler C, Domı´nguez Luzon MA, Wisplinghoff H, Rodrı´guez-Valera F (2005). Development of a multilocus sequence typing scheme for characterization of clinical isolates of *Acinetobacter baumannii*. J Clin Microbiol..

[15] Park YK, Jung SI, Park KH, Cheong HS, Peck KR, Song JH, Ko KS (2009). Independent emergence of colistin-resistant *Acinetobacter* spp. isolates from Korea. Diagn Microbiol Infect Dis.

[16] Sinha N, Agarwal J, Srivastava S, Singh M (2013). Analysis of carbapenem resistant *Acinetobacter* from a tertiary care setting in North India. Indian J Med Microbiol..

[17] Vila J, Martí S, Sánchez-Céspedes J (2007). Porins, efflux pumps and multidrug resistance in *Acinetobacter baumannii*. J Antimicrob Chemother.

[18] El-Ageery SM, Abo-Shadi MA, Alghaithy AA, Ahmad MA, Alsharif NH, Alharbi SA (2012). Epidemiological investigation of nosocomial infection with multidrug-resistant *Acinetobacter baumannii*. Eur Rev Med Pharmacol Sci..

[19] Nwadike VU, Ojide CK, Kalu EI (2014). Multidrug resistant *Acinetobacter* infection and their antimicrobial susceptibility pattern in a Nigerian tertiary hospital ICU. Afr J Infect Dis..

[20] Taneja N, Singh G, Singh M, Sharma M (2011). Emergence of tigecycline and colistin resistant *Acinetobacter baumannii* in patients with complicated urinary tract infections in North India. Indian J Med Res.

[21] Patwardhan RB, Dhakephalkar PK, Niphadkar KB, Chopade BA (2008). A study on nosocomial pathogens in ICU with special reference to mutiresistant *Acinetobacter baumannii* harboring multiple plasmids. Indian J Med Res.

[22] Ning D, De-zhi L, Ji-chao C, Yu-sheng C, Rong G, Ying-hui HU, Jing-ping Y, Juan D, Cheng-ping H, Wei Z, Jia-shu L, Qin Y, Huan-ying W, Lan M, Xiao-ning Z, Li-ping W, Jian-jun M, Qiu-yue W, Ke H, Gui-zhen T, Shao-xi C, Rui-qin W, Bei H, Si-qin W, Zhan-wei W, Su-rui Z, Zhan-cheng G (2010). Drug-resistant genes carried by *Acinetobacter baumannii* isolated from patients with lower respiratory tract infection. Chin Med J.

[23] Lean SS, Suhaili Z, Ismail S, Rahman NIA, Othman N, Abdullah FH, Jusoh Z, Yeo CC, Thong KL (2014). Prevalence and Genetic Characterization of Carbapenem- and Polymyxin-Resistant *Acinetobacter baumannii* Isolated from a Tertiary Hospital in Terengganu, Malaysia. ISRN, Microbiology..

[24] Al-Agamy MH, Khalaf NG, Tawfick MM, Shibl AMA, Kholy AE (2014). Molecular characterization of carbapenem-insensitive *Acinetobacter baumannii* in Egypt. Int J Infect Dis..

[25] Denys GA, Callister SM, Dowzicky MJ (2013). Antimicrobial susceptibility among gram-negative isolates collected in the USA between 2005 and 2011 as part of the Tigecycline Evaluation and Surveillance Trial (T.E.S.T.). Ann Clin Microbiol Antimicrob..

[26] Ertürk A, Çiçek AC, Gümüş A, Cüre E, Şen A, Kurt A, Karagöz A, Aydoğan N, Sandall Durmaz R (2014). Molecular characterisation and control of *Acinetobacter baumannii* isolates resistant to multi-drugs emerging in inter-intensive care units. Ann Clin Microbiol Antimicrob.

[27] Dettori M, Piana A, Deriu MG, Curto PL, Cossu A, Musumeci R, Cocuzza C, Astone V, Contu MA, Sotgiu G (2014). Outbreak of multidrug-resistant *Acinetobacter baumannii* in an intensive care unit. New Microbiol.

[28] Shete VB, Ghadage DP, Muley VA, Bhore AV (2010). Multi-drug resistant *Acinetobacter* ventilator-associated pneumonia. Lung India..

[29] Shete VB, Ghadage DP, Muley VA, Bhore AV (2009). *Acinetobacter* septicaemia in neonates admitted to intensive care units. J Lab Phys.

[30] Said KB, Al-Jarbou AN, Alrouji M, Al-Harbi HO. Surveillance of antimicrobial resistance among clinical isolates recovered from a tertiary care hospital in Al Qassim, Saudi Arabia. Int J Health Sci (Qassim). 2014; 8:3–12. 10.12816/0006066 PMC4039578 24899874

[31] Chang KC, Lin MF, Lin NT, Wu WJ, Kuo HY, Lin TY, Yang TL, Chen YC, Liou ML (2012). Clonal spread of multidrug-resistant *Acinetobacter baumannii* in eastern Taiwan. J Microbiol Immunol Infect.

[32] Behera B, Das A, Mathur P, Kapil A, Gadepalli R, Dhawan B (2009). Tigecycline susceptibility report from an Indian tertiary care hospital. Indian J Med Res.

[33] Spiliopoulou A, Jelastopulu E, Vamvakopoulou S, Bartzavali C, Kolonitsiou F, Anastassiou ED, Christofidou M. In vitro activity of tigecycline and colistin against *A.* *baumannii* clinical bloodstream isolates during an 8-year period. J Chemother. 2014. doi:10.1179/1973947814Y.0000000193**[Epub ahead of print]**. 10.1179/1973947814Y.0000000193 24827985

[34] Lesho E, Yoon EJ, McGann P, Snesrud E, Kwak Y, Milillo, Onmus-Leone F, Preston L, St. Clair K, Nikolich M, Viscount H, Wortmann G, Zapor M, Grillot-Courvalin C, Courvalin P, Clifford R, Waterman PE (2013). Emergence of colistin-resistance in extremely drug-resistant Acinetobacter baumannii containing a novel pmrCAB operon during colistin therapy of wound infections. J Infect Dis..

[35] Navon-Venezia S, Leavitt A, Carmeli Y (2007). High tigecycline resistance in multidrug-resistant *Acinetobacter baumannii*. J Antimicrob Chemother.

